# Preparation and Performance Evaluation of Platinum Barium Hexaaluminate Catalyst for Green Propellant Hydroxylamine Nitrate Thrusters

**DOI:** 10.3390/ma14112828

**Published:** 2021-05-25

**Authors:** Shinjae Kang, Sejin Kwon

**Affiliations:** Aerospace Engineering, Korea Advanced Institute of Science and Technology, Daejeon 34141, Korea; trumpet@kaist.ac.kr

**Keywords:** platinum, barium hexaaluminate, hydroxylamine nitrate, thruster, performance evaluation

## Abstract

Spacecraft have monopropellant thruster systems for attitude control in the vacuum of space. Hydroxylamine nitrate is a green propellant that has high performance and low toxicity. Owing to the high adiabatic decomposition temperature of the hydroxylamine nitrate propellant, it is necessary to develop a catalyst with high thermal stability. We used a platinum barium hexaaluminate catalyst for green propellant hydroxylamine nitrate thrusters. Barium hexaaluminate support was prepared by a wet impregnation method and heat treatment. Platinum, the active material, was coated on catalyst supports. The Brunauer–Emmett–Teller specific surface was also investigated. X-ray diffraction and scanning electron microscope imagery were used to confirm the formation of barium hexaaluminate. A hydroxylamine nitrate propellant blended with methanol was used for performance evaluation via firing tests of the thruster. The catalytic decomposition performance of each test was evaluated by calculating the characteristic velocity efficiency using the pressure of the chamber at the end of the catalyst bed and the mass flow rate of the propellant. As the catalyst bed was preheated to 350 °C, the characteristic velocity efficiency was 71.9%. Test results revealed that the platinum barium hexaaluminate catalyst is feasible for a hydroxylamine nitrate thruster.

## 1. Introduction

**Figure 1:**
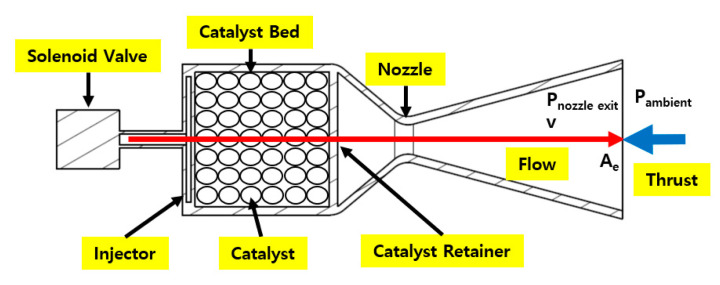
Typical monopropellant thruster.

Aircraft can maneuver in the earth’s atmosphere by using their flight control surface. Space spacecraft, such as space launch vehicles and artificial satellites, need to conduct station keeping and orbit transfer. However, there is no air in space. Unlike an aircraft, a spacecraft cannot use any flight control surface. It conducts attitude control using thrusters. For example, a space launch vehicle uses thrusters to control the attitude of its upper stage. An artificial satellite uses thrusters for station-keeping to overcome disturbances such as drag in low orbit, earth magnetic field, earth micro gravitation change, and solar wind. Many spy satellites conduct orbit transfers using their thrusters [1]. Recently, the Perseverance fired its thrusters for Mars orbit injection and landing [2]. Among the many types of thrusters for these purposes, monopropellant thrusters using one liquid propellant have various advantages, such as low complexity, high reliability, and moderate performance. A typical monopropellant thruster is fed a pressurized propellant. The catalyst bed inside the thruster decomposes the fed propellant. The decomposed propellant turns into a high-temperature gas and is ejected through a supersonic nozzle. Based on the principle of action and reaction, the thruster generates thrust (Equation (1)). Figure 1 shows a typical monopropellant thruster. $\dot{m}$ is mass flow rate, *v* is speed of gas flow, and *Ae* is the area of a nozzle exit.

(1) $$F=\dot{m}v+A_{e}\left(P_{nozzle\;exit}-P_{ambient}\right)$$

Currently, most monopropellant systems use hydrazine as a propellant; however, hydrazine is a fairly strong carcinogen and is flammable. Therefore, appropriate safety gear and facilities are required for the safety of workers and the environment [3]. With the current concern regarding the environment, studies on finding a replacement for hydrazine have been conducted. Candidates include non-toxic propellants, green propellants such as ionic liquid propellants, and hydrogen peroxide. Among ionic liquid propellants, a hydroxylamine nitrate (HAN) propellant blended with fuel has a higher specific impulse, higher density, and much lower toxicity than hydrazine [4,5,6,7]. Recently, the Green Propellant Infusion Mission of NASA was successfully completed. They used the HAN propellant AF-M315E. The technology demonstrator showed high performance and reliability in the earth’s orbit [8].

Because the HAN propellant is decomposed on the surface of a catalyst, the choice of catalyst plays an important role in determining the performance of a HAN monopropellant system. Conventional catalysts such as S405 have been reported to suffer from sintering and deactivation of the catalyst when used in HAN thrusters [9]. Therefore, a novel catalyst is required. The catalyst support should be inert in unwanted reactions, have sufficient mechanical and compressive strength to endure the harsh environment in the combustion chamber, have a large specific surface area, and have high thermal stability [10]. For a HAN monopropellant thruster, maintaining a relatively large specific surface area of a catalyst at high temperatures is important to achieve a sufficient decomposition rate at high adiabatic temperatures, above 1700 K. Therefore, catalyst supports with high thermal stability are required for HAN propellants. Barium hexaaluminate (BHA) catalyst supports for catalytic combustion processes, such as methane combustion, have shown excellent thermal stability because of their high heat resistance during sintering due to the suppression of crystal growth by large alkaline earth cations in the crystal structure [11,12]. The active center of a catalyst should provide high reactivity with a HAN propellant and endure high-temperature oxidizing conditions. In previous studies, many researchers selected iridium for a catalyst active material for HAN decomposition [4,13,14]. However, HAN is an oxidizing compound, and the iridium active center can be damaged. Platinum, another precious metal, can endure the high-temperature and high-oxidizing conditions of the combustion chamber. Previous studies have shown that HAN-water binary solutions and blended HAN propellants decompose with platinum catalysts in reactors [15,16,17]. Therefore, in this study, platinum was selected as the active catalyst material.

In this study, the catalytic decomposition performance of Pt/BHA was evaluated using a thruster firing test. For this purpose, the Pt/BHA catalyst was fabricated by a wet impregnation method on alumina pellets, and the Brunauer–Emmett–Teller (BET) specific surface area of the catalyst was investigated. X-ray diffraction and SEM analyses were performed to confirm the fabrication of the catalyst. A laboratory-produced 80 wt.% HAN aqueous solution was fabricated, and a HAN propellant blended with methanol was prepared. A HAN monopropellant thruster was designed to examine catalytic decomposition performance with a characteristic velocity efficiency. A thruster test with an 80 wt.% aqueous solution was conducted to figure out whether a decomposition reaction could be possible in a thruster environment with the Pt/BHA catalyst. After a test with 80 wt.% aqueous HAN solution by the Pt/BHA catalyst, a thruster test with the blended HAN propellant was conducted. The catalytic decomposition performance was evaluated using characteristic velocity efficiency.

## 2. Catalyst Preparation

The Pt/BHA catalyst was prepared using a wet impregnation method. The sol–gel method is commonly used to fabricate hexaaluminate catalyst supports. However, surveying a proper consolidating agent and pelletizing method for hexaaluminate powder is a complex procedure for hexaaluminate production because hexaaluminate, which is a sintering resistant material, needs to be sintered [9,11,12,18]. However, if BHA was produced by a wet-impregnation method on alumina pellets, the fabrication procedure could be much simpler. A previous study showed that barium-doped γ-Al_2_O_3_ powder subjected to a wet impregnation method could form BHA after high-temperature heat treatment [19]. Therefore, the transformation of barium-doped γ-Al_2_O_3_ pellets into BHA pellets was an effective method to obtain a simple procedure.

Alumina pellets (Alfa-Aesar) crushed to 16–20 mesh size (0.85–1.18 mm) were used in this study. A 0.25 mol/L barium nitrate (Ba(NO_3_)_2_, Sigma-Aldrich, Saint Louis, USA) solution, which was made with the required amounts of barium nitrate, and deionized water, were used to load barium on the alumina pellets. The alumina in the barium nitrate solution was dried at 105 °C in air. The catalyst support heat treatment was performed at 1000 °C in air. Subsequently, another heat treatment at 1200 °C in an air atmosphere was performed to form BHA.

**Figure 2:**
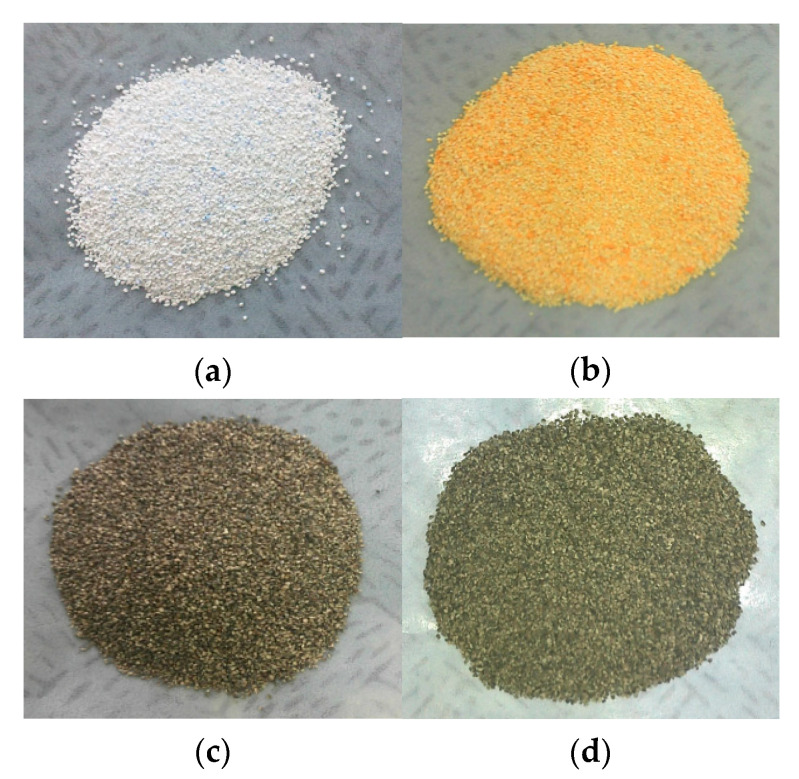
The Pt/Barium hexaaluminate (**a**) before Pt doping, (**b**) after impregnation, (**c**) after calcination, and (**d**) after reduction.

Using the wet impregnation method, platinum was loaded on the BHA catalyst support using the required amount of chloroplatinic acid (H_2_PtCl_6_∙xH_2_O, PMRESEARCH, Republic of Korea) solution as a precursor. The catalyst support was dried at 90 °C in air and then calcinated at 500 °C in air. A reduction procedure was performed to activate the platinum active center with 4% hydrogen gas (96% nitrogen gas) at 500 °C. Figure 2 shows the images of the Pt/BHA catalyst for each procedure.

**Figure 3:**
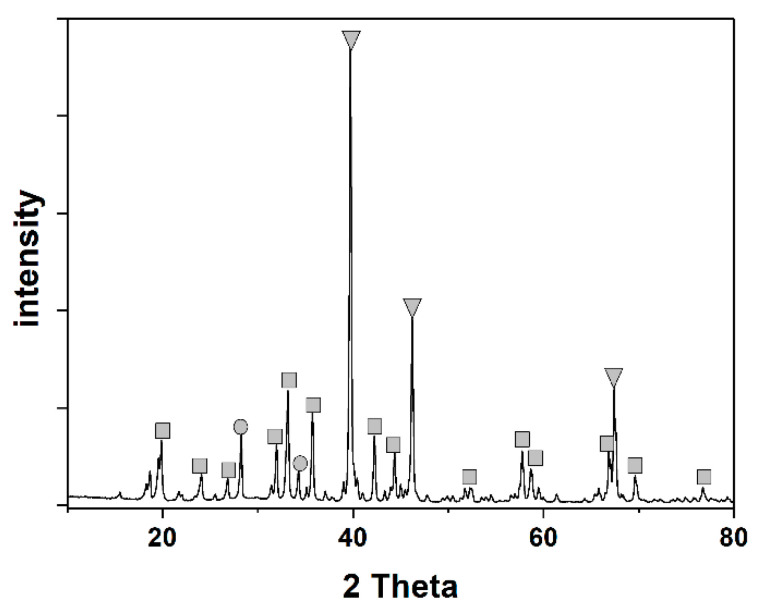
XRD analysis of the Pt/barium hexaaluminate: Ba_0.83_Al_11_O_17.33_ (■), BaAl_2_O_4_ (●), and Pt (▼).

**Figure 4:**
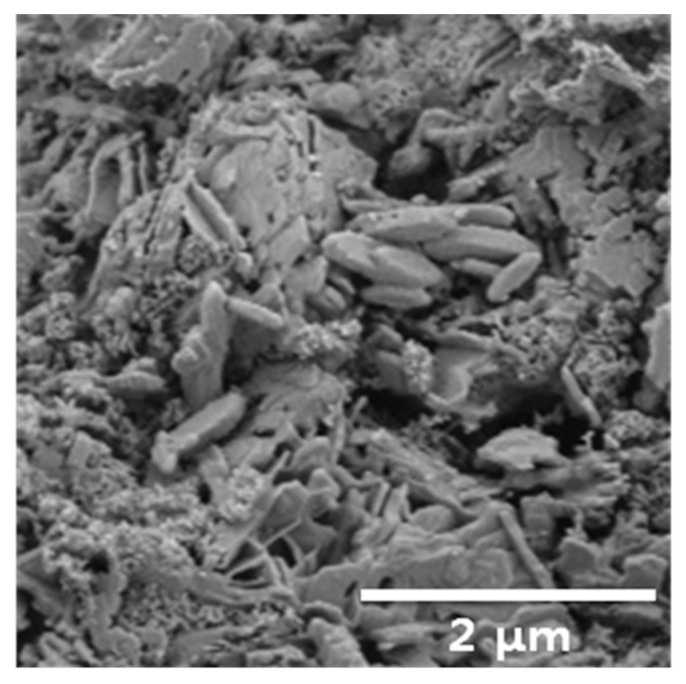
Scanning electron microscopy image of the Pt/BHA catalyst.

The BET specific surface area was measured from nitrogen absorption isotherms at 77.3 K on a Micromeritics TriStar II 3020. The Pt/BHA catalyst had 60.41 m^2^/g of BET specific surface area. Composition identification was conducted by X-ray diffraction measurements using a Rigaku D/MAX-2500 instrument at 40 kV and 300 mA. Figure 3 shows the XRD analysis results for the Pt/BHA catalyst. The XRD data showed that BHA (Ba_0.83_Al_11_O_17.33_), which suppresses the phase transformation of the catalyst support and improves thermal stability, was successfully formed in the catalyst support by the wet impregnation method and heat treatment. The loading of platinum active centers was also confirmed. Figure 4 shows an SEM image of the Pt/BHA catalyst obtained with an FEI NOVA230. The hexagonal and layered structure shown on the surface indicates the presence of BHA in the catalyst support. In magnetoplumbite-type hexagonal layered structures, anisotropic crystal growth occurs because crystal growth in a certain direction is suppressed [11,20].

## 3. Preparation for Performance Test

The 80 wt.% HAN aqueous solution was synthesized in the laboratory. The HAN aqueous solution was synthesized by mixing the required amount of 60 wt.% nitric acid (HNO_3_, OCI chemical) and 50 wt.% hydroxylamine (NH_2_OH, Aldrich). Equation (2) shows the HAN synthesis reaction between the two chemicals. The synthesized HAN could be thermally decomposed during synthesis, which is dangerous. To minimize the possibility of thermal decomposition of the HAN aqueous solution, it was maintained at a temperature below 4 °C during the synthesis.

(2) $$NH_{2}OH\;\left(aq\right)+HNO_{3}\;\left(aq\right)\underset{}{\to }\;[NH_{3}OH{]}^{+}[NO_{3}{]}^{-}\left(aq\right)$$

When the synthesis was finished, the concentration of the resulting HAN aqueous solution was 57.8 wt.%, and this was confirmed by ion chromatography. The synthesized HAN aqueous solution was concentrated by evaporating water to produce 80 wt.% HAN aqueous solution. To avoid thermal and autocatalytic reactions of the HAN solution, a rotary evaporator (RV 10V, IKA, Germany) was used for the concentration process. The HAN aqueous solution was concentrated at 33 °C under a pressure of 7 mbar. A condition for the concentration was determined by vapor pressure calculation according to Equation (3). *w* is concentration of the HAN aqueous solution in unit of weight percent.

(3) $$\mathrm{Vapor}\;\mathrm{pressure}\;\left(\mathrm{bar}\right)=\frac{\frac{100-w}{18}}{\frac{100-w}{18}+2\times \frac{w}{96}}\times 0.03136$$

The weight percentage of the HAN solution was determined by density measurements at room temperature. Equation (4) shows a formula representing the relationship between the density and weight percentage of an aqueous HAN solution [16]. The density of the 80 wt.% HAN solution was 1.514 g/mL.

(4) $$\rho =\frac{107.85}{96-30.99w}$$

The HAN aqueous solution was blended with methanol. Thrust performance, such as the specific impulse (a value from thrust divided by the propellant mass flow rate and gravitational acceleration), is improved by blending methanol. The decomposition of HAN generates oxidizing agents, as shown in Equation (5) [21] Hydrocarbon-like methanol is combusted with these oxidizing agents, and it produces high-temperature gas, as expressed in Equations (6) and (7). As the supersonic nozzle of a thruster converts thermal energy to kinetic energy, blending HAN with methanol improves the specific impulse.

(5) $$4NH_{3}OHNO_{3}\left(aq\right)\to 7H_{2}O\left(g\right)+3N_{2}O\left(g\right)+2HNO_{3}\left(g\right)$$

(6) $$6HNO_{3}\left(g\right)+5CH_{3}OH\left(aq\right)\to 13H_{2}O\left(g\right)+3N_{2}\left(g\right)+5CO_{2}\left(g\right)$$

(7) $$3N_{2}O\left(g\right)+CH_{3}OH\left(aq\right)\to 2H_{2}O\left(g\right)+3N_{2}\left(g\right)+CO_{2}\left(g\right)$$

Another advantage of the blended HAN propellant is that it can reduce the viscosity of a highly concentrated aqueous HAN solution. To achieve higher performance without methanol, the HAN aqueous solution should be highly concentrated to obtain more energetic material in an aqueous solution. However, the viscosity of HAN aqueous solution with 80 wt.% is 7.11 m Pas, which is 7–8 times higher than that of water [22]. If the HAN aqueous solution was blended with methanol, the viscosity was reduced.

Table 1 shows the adiabatic temperature and specific impulse of the methanol-blended propellant with varying amounts of methanol and water. The NASA Chemical Equilibrium Analysis (CEA) code was used for the calculations [23]. The stoichiometric ratio of the methanol-blended HAN propellant was 60 wt.%, water 26 wt.%, and methanol 14 wt.%. However, its adiabatic chamber temperature, 1802.49 K, was far above the allowable temperature of the thruster material. To fabricate a design model HAN thruster with limited funding, a lower adiabatic temperature was preferred. We chose stainless steel 316 L with an allowable temperature of 1064 K owing to its compatibility with HAN. Table 1 shows the adiabatic temperature and specific impulse (Isp) of methanol-blended propellants with varying amounts of methanol and water. To compensate for the performance and chamber temperature, the following propellant composition was selected: HAN 60 wt.%, water 30 wt.%, and methanol 10 wt.%. The density of the blended HAN propellant is 1.335 g/mL.

## 4. Thruster Setup

**Figure 5:**
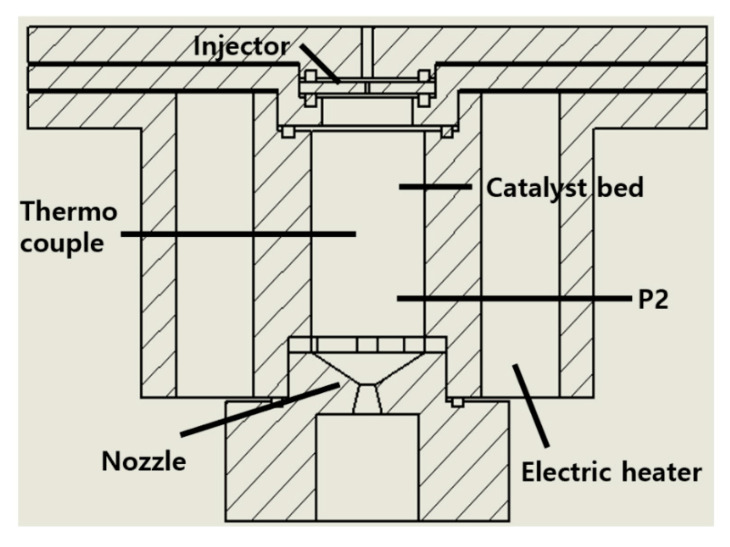
Schematic of the HAN thruster.

A HAN thruster was designed to evaluate the catalytic decomposition capability of the Pt/BHA catalyst. Figure 5 shows a schematic of the HAN thruster. The thrust level of the HAN thruster was 1.5 N, and the chamber pressure was 9 bar. The required mass flow rate was 1.04 g/s, as expressed in Equation (8). *g_o_* is gravitational acceleration.

(8) $$F=\dot{m}Ispg_{o}$$

The catalyst bed should be sufficiently large to decompose the designed mass flow rate of the propellant. However, a longer catalyst bed length led to a larger pressure drop. The propellant would not be uniformly distributed if the diameter of the catalyst bed was too large. The designed length, diameter and catalyst bed volume of the catalyst bed were 18 mm, 10 mm, and 1.675 cm^3^, respectively. We selected a single-hole injector, rather than a commercial spraying injector, to minimize the injector volume. A nozzle was designed to have optimum expansion under sea-level conditions because all thruster tests were conducted under sea-level conditions. Many thrusters operate under vacuum conditions, but the tests were conducted under sea-level conditions owing to the lack of a vacuum chamber. There is a reason why we can still conduct the tests under sea-level conditions. If the flow speed exceeds the sonic speed of the gas in a supersonic nozzle, the pressure at the nozzle exit cannot influence the upper stream of the nozzle. Therefore, the conditions inside the catalyst bed were the same regardless of the nozzle exit conditions. Therefore, we conducted performance evaluation tests under sea-level conditions. The geometry of the nozzle was that of a 15-degree cone nozzle.

Pressure ports were placed to measure chamber pressure (P2). The P2 pressure port was assumed to be the end of the catalyst bed. Owing to the small volume of the catalyst bed, the position of P2 was the nearest position to the end of the catalyst bed that we could use. The catalyst bed of the HAN thruster needed to be preheated for the decomposition of a given amount of propellant, although a precious metal catalyst was used [5]. The metal-sheltered electric heater, which was used for the hydrazine thruster with high reliability, exhibited uniform heat transfer, high thermal conductivity, and electrical insulation at high temperatures. The performance of the fabricated heater with Inconel 600 was 110 V and 50 W, and four heaters were used. The temperature was measured in the middle of the catalyst bed to determine the preheating temperature.

**Figure 6:**
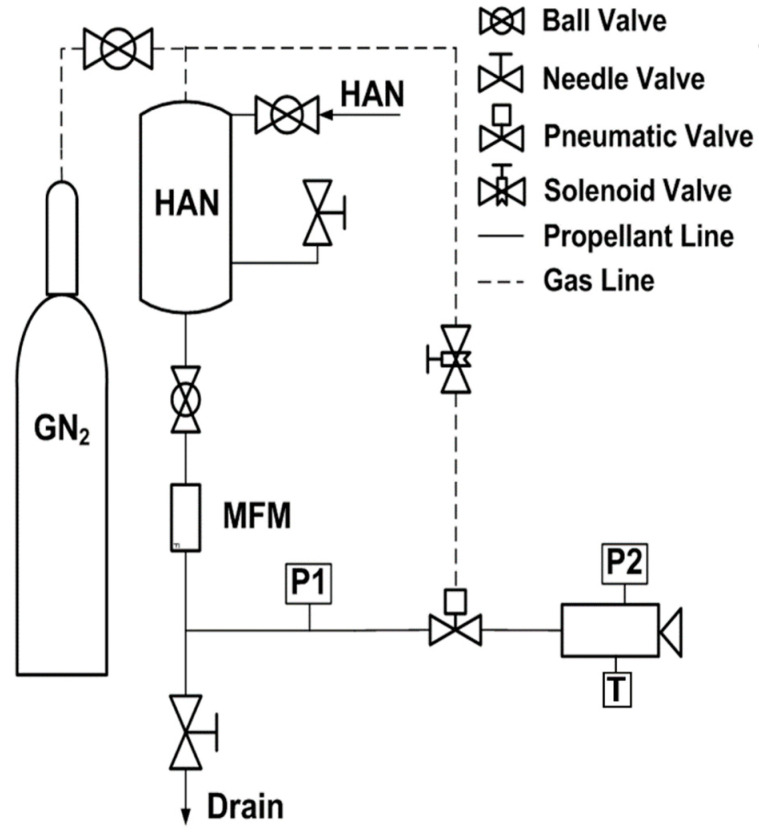
Schematic of the experimental setup.

Figure 6 shows a schematic of the experimental thruster setup. All parts were made of stainless steel 316 owing to its compatibility with HAN. The propellant was pressurized using high-pressure nitrogen gas. The mass flow rate of the propellant was measured using an orifice plate (D and D/2 tap type) mass flow meter. The propellant was supplied through a pneumatic valve (Swagelok) controlled by a solenoid valve. The timer mechanism controls the opening and closing of the valve. The pressure data and propellant mass flow rate were recorded by National Instruments at a data acquisition rate of 100 Hz with a 10 kHz filter.

## 5. Performance Evaluation by Thruster Firing Test

### 5.1. Evaluation Method

The characteristic velocity efficiency was selected to examine the catalytic decomposition performance. The characteristic velocity efficiency can indicate the degree of propellant decomposition [24]. A high characteristic velocity implies that most of the energy of the propellant was released, and high-temperature gas was created (Equation (9)). We calculated the theoretical characteristic velocity using the NASA CEA code. Through Equation (10), we calculated the empirical characteristic velocity efficiency using the measured chamber pressure, propellant mass flow rate, and nozzle throat area. The characteristic velocity efficiency was calculated using Equation (11). *k* is the specific heat ratio, *R* is the gas constant, *T* is the gas temperature, and *A_t_* is the area of the nozzle throat.

(9) $$c_{theo}^{*}=\frac{\sqrt{kRT}}{k\sqrt{{\left[2/\left(k+1\right)\right]}^{\left(k+1\right)/\left(k-1\right)}}}$$

(10) $$c_{exp}^{*}=\frac{P_{2}A_{t}}{\dot{m}}$$

(11) $$\eta_{C^{*}}=\frac{c_{exp}^{*}}{c_{theo}^{*}}\cdot 100$$

### 5.2. Preliminary Test with 80 wt.% HAN Aqueous Solution

**Figure 7:**
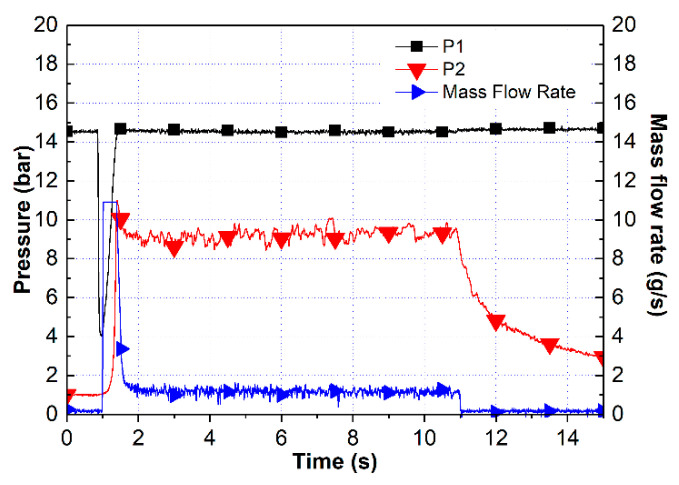
Test result for the 80 wt.% HAN aqueous solution.

An 80 wt.% HAN aqueous solution was used to examine whether HAN could be decomposed in the combustion chamber environment by the Pt/BHA catalyst. In the case of the HAN thruster, the catalyst bed should be preheated for the complete decomposition of the propellant. Therefore, the catalyst bed was preheated to 300 °C. The preheating temperature was measured in the middle of the catalyst bed. The 80 wt.% HAN aqueous solution was injected into the thruster chamber for 10 s, and the feeding pressure was 15 bar. Figure 7 shows the pressure and mass flow rates during the thruster test. The mass flow rate of the propellant was 1.31 g/s, and the chamber pressure was 9.22 bar. At a theoretical characteristic velocity of 80 wt.% HAN aqueous solution of 713 m/s, the characteristic velocity efficiency was 77.1%. As shown in Figure 7, we measured the stable chamber pressure and mass flow rate. With the characteristic velocity efficiency, the Pt/BHA catalyst could decompose HAN in the thruster chamber environment.

We discovered that the catalyst bed should be preheated at a higher temperature in the thruster test than in the reactor tests. In the study by Courtheoux et al., with a constant volume batch reactor, 79 wt.% aqueous solution was decomposed at 56 °C in the presence of a platinum catalyst [16]. In a study by Armarier et al., 80 wt.% HAN aqueous solution showed catalytic decomposition at 77 °C with a platinum catalyst in a dynamic reactor test [17]. In these studies, HAN could require a much longer time to be absorbed and decomposed on the surface of the catalyst than in the case of the thruster test. In our thruster test, the HAN needed to be decomposed at a much faster rate owing to the short residence time in the catalyst bed. Therefore, a preheating temperature of at least 300 °C is required.

After the test, the Pt/BHA catalyst was severely damaged. Then, it was crushed into a powder state. The copper ring used for sealing was corroded by nitric acid. The decomposition reaction of HAN solution (Equation (5)) generated a high concentration of nitric acid, which weakened the structure of the alumina and made the alumina vulnerable in the thruster chamber environment. The alumina catalysts in the thruster chamber weakened by nitric acid generated in the decomposition could be crushed due to instantaneous high temperature and pressure build-up by hot gases in the pores of the alumina catalysts. This phenomenon has a negative impact on the durability of the alumina catalyst and causes a pressure drop in the thruster chamber.

### 5.3. Performance Evaluation Test with 300 °C Preheating Temperature of the Catalyst Bed

The catalyst bed was preheated at 300 °C, the same temperature as that used in the thruster test with 80 wt.% HAN aqueous solution. As the adiabatic decomposition temperature of the blended HAN propellant was 1453 K, the operation time was set to 5 s to avoid thermal problems caused by the high temperature of the decomposed gas, and the feeding pressure was 15 bar.

**Figure 8:**
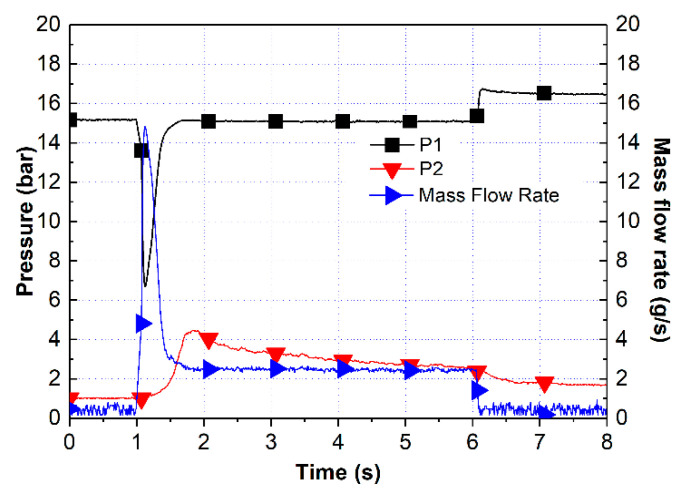
Test result for the Pt/BHA catalyst with preheated catalyst bed at 300 °C.

The results of the thruster tests with the Pt/BHA catalyst are shown in Figure 8. The measured mass flow rate of the blended HAN propellant was 2.47 g/s, and the maximum chamber pressure was 4.47 bar. As the theoretical characteristic velocity of the blended HAN propellant was 1144.2 m/s, the characteristic velocity efficiency was 19.2%. A lower decomposition rate was observed visually during the thruster test. The majority of the blended HAN propellant was not decomposed and was flushed out of the catalyst bed. Therefore, the tests with the blended HAN propellant showed lower decomposition rates, and the catalyst bed should be preheated to a higher temperature, such as 350 °C, to achieve a higher decomposition rate.

Compared with the results obtained with 80 wt.% HAN aqueous solution, the characteristic velocity efficiency was significantly decreased. The lower characteristic velocity efficiency of the tests with the blended HAN propellant may be due to the composition of the blended HAN propellants with 60 wt.%, H_2_O 30 wt.%, and methanol 10 wt.%. The entire reaction in the catalyst bed started with the HAN decomposition reaction expressed in Equation (5). To initiate the catalytic decomposition of HAN, HAN dissolved in water was adsorbed in the active centers on the surface of the catalyst. The blended HAN propellant had a smaller chance of being absorbed in the active centers than the 80 wt.% HAN aqueous solution because of the lower concentration of HAN molecules. In addition, the HAN molecules had to compete with methanol molecules for the active centers. This was the reason why the test with the blended HAN propellant showed low characteristic velocity efficiency.

### 5.4. Performance Evaluation Test with 350 °C Preheating Temperature of the Catalyst Bed

The catalytic decomposition performance of the Pt/BHA catalyst was evaluated when the catalyst bed was preheated to 350 °C. The blended HAN propellant was fed into the reaction chamber for 5 s, as in the previous tests, and the feeding pressure was maintained at 15 bar.

**Figure 9:**
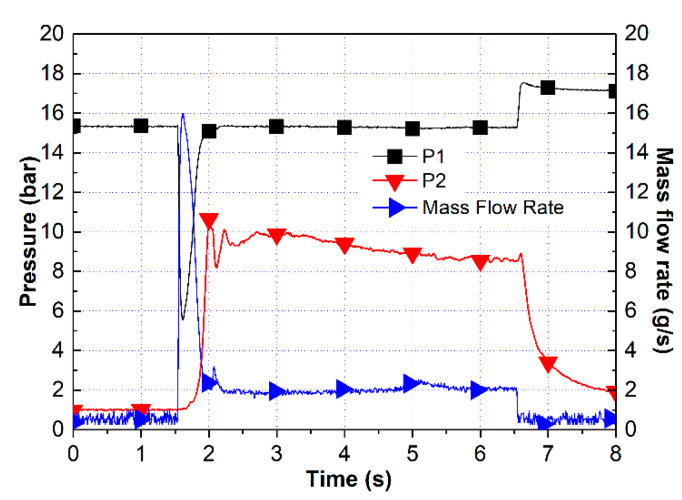
Test result for the Pt/BHA catalyst with the preheated catalyst bed at 350 °C.

Figure 9 shows the measured pressure of the Pt/BHA catalyst. The mass flow rate of the blended HAN propellant was 2.04 g/s. The characteristic velocity efficiency of Pt/BHA was 71.9%, which was better than that of the previous test. This implies that the Pt/BHA catalyst could decompose more of the blended HAN propellant in the reaction chamber.

The performance evaluation of the Pt/BHA catalyst showed its feasibility for HAN monopropellant thruster applications. As described in the Introduction section, a catalyst for a HAN monopropellant thruster should be thermally stable at high temperatures. To fabricate a thermally stable catalyst, some of the BET specific surface areas were sacrificed as the Pt/BHA catalyst. The Pt/BHA catalyst could maintain the BET specific surface area by doping with barium and creating a hexaaluminate form. Therefore, the catalyst could provide a sufficient surface for the decomposition of the HAN propellant. The platinum active material provided the decomposition capability of the HAN propellant in the thruster environment. Certainly, a characteristic velocity efficiency of approximately 70% is insufficient for the real application of the HAN thruster. Typically, commercial monopropellant thrusters have a characteristic velocity efficiency of above 92%. However, this test was designed to determine the feasibility of the Pt/BHA catalyst for HAN thruster applications. With a higher preheating temperature above 400 °C and a larger volume of the catalyst bed, the characteristic velocity efficiency of a thruster with the Pt/BHA catalyst could reach above 90%. Therefore, one can say that the Pt/BHA catalyst could be used for HAN monopropellant thruster applications.

The elevated preheating temperature increased the reactivity of the Pt/BHA catalyst. The Arrhenius equation is a common explanation of the catalyst reactivity, as expressed in Equation (12). The Arrhenius equation allows a catalyst with an elevated surface temperature to have a higher reactivity. As the reactivity of Pt/BHA increased, a greater amount of the blended HAN propellant was decomposed.

(12) $$\mathrm{Rate}\;\mathrm{constant}=Ae^{-E_{a}/RT}$$

The Pt/BHA catalyst was not damaged when the blended HAN propellant was used. This implies that the methanol in the blended HAN propellant consumes nitric acid generated in the HAN decomposition reaction (Equation (6)), and a negative impact caused by nitric acid did not occur. Therefore, the Pt/BHA catalyst could retain its shape.

## 6. Conclusions

The catalytic decomposition performance of the Pt/BHA catalyst was evaluated using the HAN thruster. Owing to the high adiabatic temperature of HAN propellants, a catalyst that can withstand high temperatures, high pressures and oxidizing conditions is required. Loading and heat treatment procedures for fabricating Pt/BHA have been established. The Pt/BHA catalyst was examined using XRD, BET, and SEM. The XRD test showed a barium hexaaluminate crystal, and the SEM image showed a hexagonal and layered structure, indicating the presence of barium hexaaluminate. The BET specific surface areas were measured.

To produce the propellant for the thruster test, the setup for HAN synthesis was designed to mix a hydroxylamine aqueous solution and nitric acid. Next, a vacuum concentration process was carried out to obtain an accurate composition of the HAN aqueous solution. The proposed composition of the blended HAN propellant is 60 wt.%, water 30 wt.%, and methanol 10 wt.%, in consideration of the allowable temperature of material in the structure of the thruster.

The thruster was designed for the performance evaluation test by hot firing tests, and its data were evaluated using the characteristic velocity efficiency. The preliminary thruster firing test was conducted using 80 wt.% HAN aqueous solution. The results showed that the characteristic velocity efficiency was approximately 80%. Therefore, the platinum catalyst could decompose HAN in both dynamic and batch reactors, as well as in a thruster. Subsequently, we evaluated the performance of the Pt/BHA catalyst by a thruster firing test using the blended HAN propellant. When the preheating temperature of the catalyst bed was 300 °C, the characteristic velocity efficiency was below 20%. This implies that the catalyst was unable to decompose a given mass flow rate of the propellant, and the blended HAN propellant was flushed through the catalyst bed. As the preheating temperature of the catalyst bed was increased to 350 °C, the measured characteristic velocity efficiency of the Pt/BHA catalyst was 71.9%. The Pt/BHA catalyst could decompose the blended HAN propellant with an elevated preheating temperature. This implies that there is potential for the Pt/BHA catalyst if a more elevated catalyst bed preheating temperature and larger volume of the catalyst bed are applied.

## Figures and Tables

**Table 1:** Adiabatic temperature and specific impulse of methanol-blended HAN propellants.

Composition (HAN:H_2_O:MeOH)	Adiabatic Temperature (K)	* Isp (s)
60:30:10	1453	207.2
60:28:12	1667	224.0
60:26:14	1802	234.6
60:24:16	1791	235.3
60:22:18	1779	236.1

* Vacuum specific impulse was calculated at a nozzle expansion ratio of 50.

## Data Availability

Not applicable.
